# Plastidial Phosphoglucomutase (*pPGM*) Overexpression Increases the Starch Content of Transgenic Sweet Potato Storage Roots

**DOI:** 10.3390/genes13122234

**Published:** 2022-11-28

**Authors:** Yannan Wang, Huan Zhang, Yan Li, Qian Zhang, Qingchang Liu, Hong Zhai, Ning Zhao, Yufeng Yang, Shaozhen He

**Affiliations:** 1 Cereal Crops Research Institute, Henan Academy of Agricultural Sciences, Zhengzhou 450002, China; 2 Key Laboratory of Sweet Potato Biology and Biotechnology, Ministry of Agriculture and Rural Affairs/Beijing Key Laboratory of Crop Genetic Improvement/Laboratory of Crop Heterosis & Utilization and Joint Laboratory for International Cooperation in Crop Molecular Breeding, Ministry of Education, College of Agronomy & Biotechnology, China Agricultural University, Beijing 100193, China; 3 College of Agronomy and Biotechnology, Southwest University, Chongqing 400715, China; 4 Provincial Key Laboratory of Agrobiology, Institute of Food Crops, Jiangsu Academy of Agricultural Sciences, Nanjing 210014, China; 5 Cereal Crops Research Institute, Henan Academy of Agricultural Sciences, Postgraduate T&R Base of Zhengzhou University, Zhengzhou 450002, China; 6 School of Agricultural Sciences, Zhengzhou University, Zhengzhou 450001, China

**Keywords:** sweet potato, *IbpPGM*, chloroplast localization, overexpression, increased starch content, altered soluble sugar levels

## Abstract

Sweet potato (Ipomoea batatas), an important root crop, has storage roots rich in starch that are edible and serve as a raw material in bioenergy production. Increasing the storage-root starch contents is a key sweet potato breeding goal. Phosphoglucomutase (PGM) is the catalytic enzyme for the interconversion of glucose-6-phosphate and glucose-1-phosphate, precursors in the plant starch synthetic pathway. Plant PGMs have plastidial and cytosolic isoforms, based on their subcellular localization. Here, *IbpPGM*, containing 22 exons and 21 introns, was cloned from the sweet potato line Xu 781. This gene was highly expressed in the storage roots and leaves, and its expression was induced by exogenous sucrose treatments. The mature IbpPGM protein was successfully expressed in *Escherichia coli* when a 73-aa chloroplastic transit peptide detected in the N-terminus was excised. The subcellular localization confirmed that IbpPGM was localized to the chloroplasts. The low-starch sweet potato cultivar Lizixiang *IbpPGM*-overexpression lines showed significantly increased starch, glucose, and fructose levels but a decreased sucrose level. Additionally, the expression levels of the starch synthetic pathway genes in the storage roots were up-regulated to different extents. Thus, *IbpPGM* significantly increased the starch content of the sweet potato storage roots, which makes it a candidate gene for the genetic engineering of the sweet potato.

## 1. Introduction

Starch is the main storage form of carbohydrates in plants. During the day, leaves synthesize transitory starch in the chloroplasts through photosynthesis. It is then degraded into sucrose at night and transported to non-photosynthetic organs as an energy source [1,2]. ADP-glucose (ADPG) is the transitory starch substrate in chloroplasts and is synthesized by the successive catalysis of plastidial glucose phosphate isomerase, phosphoglucomutase (PGM) and ADP-glucose pyrophosphorylase. The ADPG is then transformed into amylose or amylopectin by the starch synthases [3].

The interconversion between glucose-1-phosphate (G1P), the precursor of ADPG, and glucose-6-phosphate (G6P) is catalyzed by PGM. Two types of PGM exist in plants, cytosolic and plastidial, and they play synergistic roles in the distribution of photosynthetic carbohydrates [4]. In plant cells, PGM maintains the equilibrium between starch and sucrose by participating in three catalytic steps. First, pPGM produces G1P from G6P, which is generated through photosynthesis in autotrophic tissues or is imported into heterotrophic tissues (amyloplasts) from the cytoplasm [5]. Second, pPGM generates G6P from the starch phosphorolysis-derived G1P [6,7]. Third, cPGM is involved in sucrose synthesis by converting G6P to G1P in the cytoplasm [8]. To date, only one pPGM and one cPGM have been identified in potato, spinach and pea [9,10,11], whereas one pPGM and two cPGM exist in *Arabidopsis*, tobacco and maize [12,13,14].

The functions of *PGM* have been characterized in different plant species. In the leaves of the *pPGM*-deficient *Arabidopsis* and tobacco mutants, the starch levels are barely detectable, but the soluble sugar levels increase [12,13,15]. A mutation of the *rug3* locus encoding pPGM in the pea results in the decreased starch contents in the leaves and seeds, which demonstrates that G6P is the source of phosphohexose in pea amyloplasts [10,16]. The inhibition of *pPGM* or *cPGM* genes in the potato reduces the photosynthetic rate in the leaves and the starch content in the tubers [17,18]. The *pPGM* mutants of *Arabidopsis* also show a photosynthetic inhibition, whereas there is no change in the photosynthetic rate of the transgenic tobacco overexpressing *AtpPGM* or *AtcPGM* [4,12]. Although PGM plays important roles in the distribution of the photosynthetic carbon metabolites, understanding how it affects the photosynthetic rate requires further study. Additionally, the loss of PGM functions may lead to the gametophyte abortion, possibly owing to an inadequate carbon-energy supply to the reproductive organs [5,19,20]. Therefore, regulating the starch or sucrose metabolic pathway in pollen is an effective method to induce male sterility, which is widely used in breeding practices [20,21].

There is increasing evidence of alternative starch synthetic pathways in plants, such as ADPG, acting as a starch synthesis substrate, being directly catalyzed by the sucrose synthase in the cytoplasm and transported to the chloroplasts [2,3,22,23]. The inhibition of the potato *pPGM* or *cPGM* expression results in a decreased tuber starch content, but no significant difference was found in the tuber starch content between the transgenic and wild-type (WT) potato when both genes are inhibited, simultaneously. This indicates that starch-synthesis precursors (UDPG, ADPG and G1P) are transported to the heterotrophic organs to replace G6P [16,24,25]. Therefore, in addition to the catalytic conversion of G6P and G1P by PGM in plastids, the transport pathways exist in the plastid membranes of autotrophic and heterotrophic organs to provide the precursors for the starch synthesis in plastids.

Sweet potato (Ipomoea batatas) is an important crop with starch-enriched storage roots that act as both a staple food source and a bio-energy raw material. The functions of the key genes in the starch synthesis have been reported in sweet potato [26,27,28,29,30,31,32,33], but the function of the *PGM* gene remains unclear. In this study, a plastidial *PGM* gene, *IbpPGM*, was cloned from the sweet potato, and its expression profiles were analyzed. The overexpression of *IbpPGM* in the sweet potato significantly altered the storage-root carbohydrate content, and the underlying mechanism is discussed.

## 2. Materials and Methods

### 2.1. Plant Materials and Reagents

The sweet potato line Xu 781, cultivar Lizixiang, tobacco (*Nicotiana benthamiana*) plants, vectors (pET-28a, pMDC83, pCAMBIA3301 and pBI121) and *Agrobacterium tumefaciens* EHA105 competent cells were kindly provided by the Key Laboratory of Sweet Potato Biology and Biotechnology, Ministry of Agriculture of China. Xu 781 was used for the *IbpPGM* isolation, and Lizixiang was used for the gene’s functional characterization.

### 2.2. Cloning and the Sequence Analysis of IbpPGM

The total RNA was extracted from the leaves of Xu 781, using a RNAprep Pure Plant Kit (Tiangen, Beijing, China) and then transcribed it into first-strand cDNA using a PrimeScript II 1st Strand cDNA Synthesis Kit (TaKaRa, Beijing, China). A pair of degenerate primers (DF/R) was designed on the basis of the most homologous region identified in a multiple alignment of *pPGM* genes from different plant species, and an expressed sequence tag fragment was amplified. Then, the rapid amplification of the cDNA ends (RACE) was conducted to obtain the full-length cDNA. The genomic *IbpPGM* sequence was amplified with primers GF/R using genomic DNA extracted from the leaves of Xu 781 as the template. The online tools ORFfinder (https://www.ncbi.nlm.nih.gov/orffinder/, accessed on 13 March 2019), ExPASy (https://web.expasy.org/compute_pi/, accessed on 27 March 2019), TargetP-2.0 (http://www.cbs.dtu.dk/services/TargetP/#, accessed on 3 April 2019) and Gene Structure Display Server (GSDS, http://gsds.gao-lab.org/, accessed on 25 May 2020) were used to analyze the open reading frame (ORF), the protein molecular weight, signal peptide, phylogenic tree and gene structure, respectively. A multiple sequence alignment between IbpPGM and other PGM proteins was conducted using DNAMAN software. The sequences of the primers used in this study are listed in Table 1.

### 2.3. Expression Analysis of IbpPGM in the Sweet Potato

The total RNA was isolated from five different tissues (storage root, fibrous root, stem, leaf, and petiole) of Xu 781 plants grown in the field for approximately 100 days, and the first-strand cDNA was synthesized using PrimeScriptTM RT Reagent Kit with gDNA Eraser (Perfect Real Time) (TaKaRa, Beijing, China). The qRT-PCR was conducted on a 7500 Real-Time PCR System (Applied Biosystems, Waltham, MA, USA) to determine the *IbpPGM* transcript levels using the gene-specific primers qPGM-F/R. The control, *IbActin*, was amplified using the primers Actin-F/R.

The Xu 781 plants that were grown in the field for approximately one month, were used to investigate the response of *IbpPGM* to exogenous sucrose. Briefly, the leaf-petioles (10 cm) of the Xu 781 plants were cultured in water in darkness for 1 d as a starvation treatment. Then, they were supplied with water or 175 mmol L^−1^ sucrose in darkness at 28 °C. The qRT-PCR was conducted to determine the transcript levels of *IbpPGM* in the cuttings harvested at different time points (0, 2, 4, 6, 12, 24 and 48 h) after treatment. Three cuttings were used as biological replicates per time point for each treatment.

### 2.4. Prokaryotic Expression of IbpPGM

To determine whether *IbpPGM* encodes a mature protein, the gene was expressed in *E. coli*. The full-length ORF and the signal peptide-cleaved ORF (ΔORF) of *IbpPGM* were amplified with primers pET-F/R and pET-ΔF/R, respectively. The sequence-verified fragments were ligated independently into the expression vector pET-28a. Then, the recombinant vectors pET-28a-IbpPGM and pET-28a-ΔIbpPGM, as well as the pET-28a native vector, were introduced independently into the competent *E. coli* strain *Transetta* (DE3) cells (Transgen, Beijing, China). Fresh Luria-Bertani medium was inoculated independently with the positive clones, and they were cultured at 28 °C until the OD600 values reached 0.8. The soluble cytoplasmic proteins were prepared from the isopropyl β-D-thiogalactopyranoside-induced *Trans*etta (DE3) cells. Then, the expressed IbpPGM protein was subjected to an SDS-PAGE analysis.

### 2.5. Subcellular Localization

The ORF of *IbpPGM* was amplified using primers 83-F/R and inserted into the pMDC83 vector between *Pac*I and *Asc*I cleavage sites. The recombinant vector pMDC83-IbpPGM and the native vector were introduced independently into the *A. tumefaciens* strain EHA105, and the positive strains were injected independently into the *N. benthamiana* leaf epidermal cells for the transient expression. Following the co-cultivation at 28 °C for approximately 36 h, the agroinfiltrated tobacco leaves were visualized using a laser scanning confocal microscope.

### 2.6. Production of the Transgenic Plants

To construct the overexpression vector, the sequence-verified ORF of *IbpPGM*, which had been amplified using primers OPGM-F/R, was inserted into pBI121 between *BamH*I and *Sac*I to replace the glucuronidase (*gus*A) gene. Subsequently, the expression cassette 35S-IbpPGM-NOS was excised from the pBI121-IbpPGM vector using *Hind*III and *Pst*I and then ligated between the same cleavage sites in pCAMBIA3301, to generate the recombinant overexpression vector pC3301-121-IbpPGM. This plasmid was transfected into the *A. tumefaciens* strain EHA105. The plant transformation and regeneration were performed, as described by Wang et al. [32] using embryogenic suspension cultures of Lizixiang established, as described by Liu et al. [34]. The putatively transgenic sweet potato plants were identified by histochemical GUS assays and PCR. Then, the positive transgenic lines were subjected to the qRT-PCR using primers qPGM-F/R, and the three lines with the highest *IbpPGM* expression levels were selected for further phenotypic analyses.

### 2.7. Quantification of the Carbohydrate Contents

The starch contents in the storage roots of the transgenic and WT plants were analyzed in accordance with the method of Smith and Zeeman [35], and high performance liquid chromatography (HPLC) was used to determine the sucrose, glucose, and fructose contents in the storage roots with the following method. First, 30 mg of 80 °C dried storage roots was dissolved in 0.7 mL of 80% ethanol to extract the sugars. Then, the sample was thoroughly vortexed and incubated for 2 h at 70 °C. The aliquots of 0.7 mL of HPLC-grade water and 0.7 mL chloroform were added to the sample. Then, after shaking several times, the mixtures were centrifuged at 12,000 g for 10 min. Then, 0.7 mL of the aqueous supernatant was transferred into 1.5-mL Eppendorf tubes and resuspended in 0.7 mL of chloroform. Following the centrifugation at 12,000 g for 10 min, 0.5 mL of the supernatant was transferred to a glass tube for the HPLC analysis of each sugar component. The Agilent technologies HPLC column (ZORBAX Carbohydrate column; 4.6 × 150 mm, 5 μm) with a differential refraction detector was used. The mobile phase consisted of 75% acetonitrile with a flow rate of 0.8 mL min**^−1^** and the temperature of the column was maintained at 35 °C. The sugars were identified, based on the retention time of the standards, and the sample concentrations were calculated from the external standard curve. The quantifications were carried out with three replicates for each plant line.

### 2.8. Expression Analysis of the Starch Biosynthetic Genes

The transcript levels of 12 key genes in the starch biosynthetic pathways of the transgenic and WT storage roots were investigated using qRT-PCR. The 12 *Ipomoea batatas* genes were *AGP-sTL1* and *2* (encoding the two small subunits of IbAGPase), *AGP-TLI* (encoding the large subunit of IbAGPase), *granule-bound starch synthase I* (GBSSI), *soluble starch synthase I* (*SSI*), *SSII*, *SSIII*, *SSIV*, *starch branching enzymes I* and *II* (*SBEI* and *SBEII*), *isoamylase1* (*ISA1*), and *pullulanase* (*PUL*). The primers used to amplify these genes are listed in Table 1.

## 3. Results

### 3.1. Cloning and Sequence Analysis of IbpPGM

**Figure 1:**
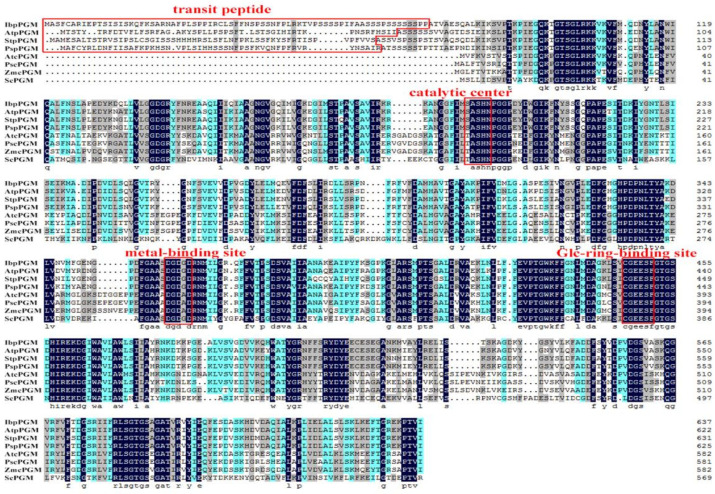
Multiple sequence alignment of PGM proteins. AtpPGM: *Arabidopsis thaliana*, AF216580; StpPGM: *Solanum tuberosum*, NM_001288352; PspPGM: *Pisum sativum*, AJ250770; AtcPGM: *A. thaliana*, At1g23190; PscPGM: *P. sativum*, AJ250769; ZmcPGM: *Zea mays*, U89341; ScPGM: *Saccharomyces cerevisiae*, NP_012795. Homology in amino acids is shaded with dark blue (homology = 100%), lightblue (homology ≥ 75%) or grey (homology ≥ 50%).

**Figure 2:**
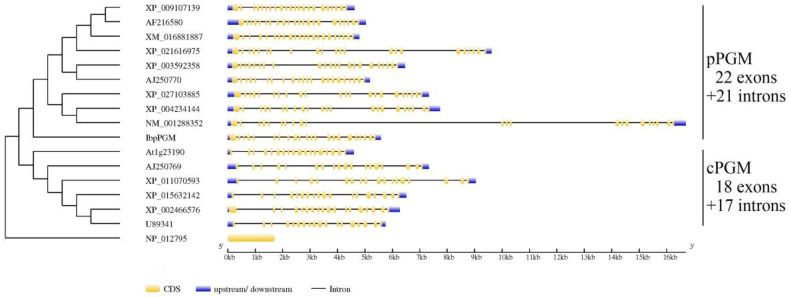
Phylogenetic and gene structure analyses of IbpPGM and PGMs from other species using GSDS. *Brassica rapa*, XP_009107139; *A. thaliana*, AF216580; *Gossypium hirsutum*, XM_016881887; *Manihot esculenta*, XP_021616975; *Medicago truncatula*, XP_003592358; *P. sativum*, AJ250770; *Coffea arabica*, XP_027103885; *Solanum lycopersicum*, XP_004234144; *S. tuberosum*, NM_001288352; *A. thaliana*, At1g23190; *P. sativum*, AJ250769; *Sesamum indicum*, XP_011070593; *Oryza sativa*, XP_015632142; *Sorghum bicolor*, XP_002466576; *Z. mays*, U89341; *S. cerevisiae*, NP_012795.

The RACE method was used to clone IbpPGM from the Xu 781 sweet potato line. The cloned 2182-bp full-length IbpPGM cDNA contained a 1917-bp ORF that generated a 638-aa protein with a molecular weight of 69.3 kDa. The genomic sequence of IbpPGM was 5583 bp and contained 22 exons and 21 introns. TargertP 2.0 and ChloroP 1.1 predicted that the N-terminus of the IbpPGM protein contains a 73-aa chloroplast transit peptide. The molecular weight of IbpPGM without the transit peptide was 61.7 kDa. A multiple-sequence alignment of the PGM protein showed that IbpPGM shared a conserved domain similar with those from several other plants and from Saccharomyces cerevisiae (Figure 1). Additionally, pPGM had a chloroplast transit peptide that was not found in cPGM. A GSDS analysis revealed that pPGM and cPGM evolved into two different branches, with members of each branch having identical exon-intron structures, and IbpPGM was most closely related to the pPGM from Solanum lycopersicum (80.82% homology) (Figure 2).

### 3.2. Expression of IbpPGM in the Sweet Potato

**Figure 3:**
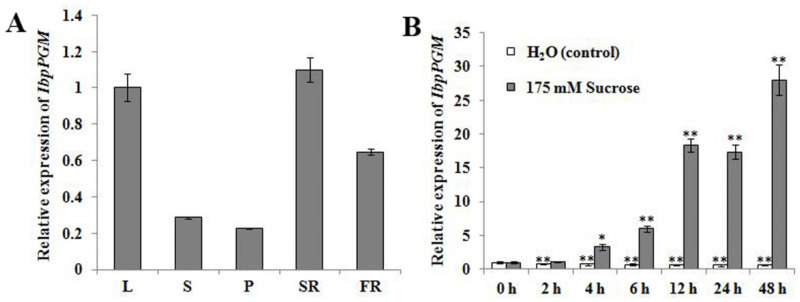
Expression patterns of *IbpPGM.* (**a**) Expression of *IbpPGM* in different tissues of Xu 781 sweet potato. L, leaves, S, stems, P, petioles, SR, storage roots, FR, fibrous roots. (**b**) Induced expression of *IbpPGM* by a 175-mM sucrose treatment. * and ** indicate a significant difference versus 0 h at *p* < 0.05 and < 0.01, respectively, based on Student’s *t*-test.

The qRT-PCR revealed that *IbpPGM* was expressed in all five tested tissues of Xu 781 plants, with the highest expression occurring in the storage root, followed, successively, by the leaves, the fibrous roots, the stems, and the petioles (Figure 3a). The 175-mM sucrose treatments of the leaf-petiole cuttings in darkness significantly induced the *IbpPGM* expression, which strongly increased at 12 h after treatment and peaked after 48 h, reaching approximately 28 times the value at 0 h (Figure 3b).

### 3.3. Expression of IbpPGM in E. coli

**Figure 4:**
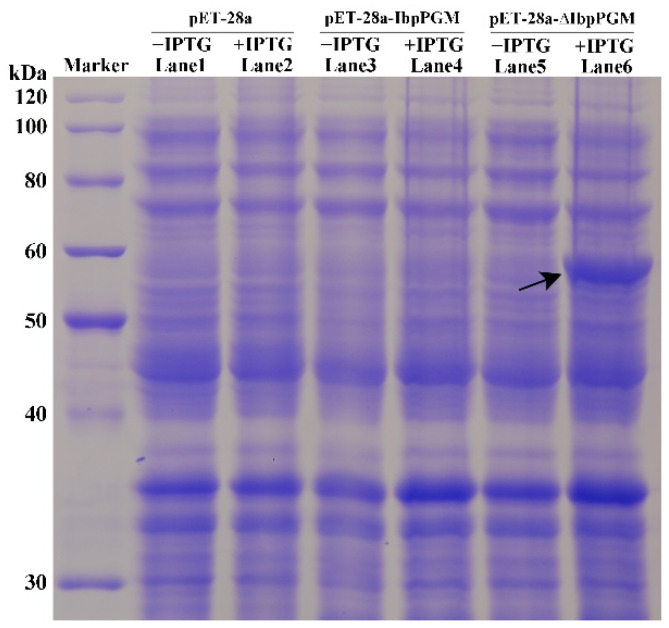
Prokaryotic expression of *IbpPGM* in *E. coli.* The arrow indicates the induced protein of IbpPGM without the chloroplast transit peptide.

To investigate whether *IbpPGM* encodes the mature protein, we constructed a recombinant vector harboring the full-length ORF of *IbpPGM*, as well as one carrying the ORF with the transit peptide removed (Δ*IbpPGM*), to eliminate the effect of the chloroplast transit peptide on the prokaryotic expression. These two vectors were then expressed independently in *E. coli* (Figure 4). The full-length ORF of *IbpPGM* failed to express the corresponding 69.3-kDa protein, whereas Δ*IbpPGM* expressed a protein of approximately 61.7 kDa. This suggested that the chloroplast transit peptide inhibits the expression of *IbpPGM* in *E. coli*.

### 3.4. Subcellular Localization of IbpPGM in N. benthamiana

**Figure 5:**
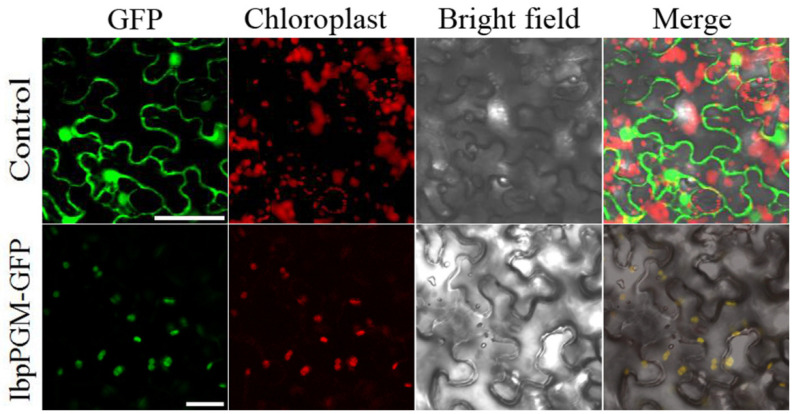
Subcellular localization of the IbpPGM protein in the epidermal cells of *N. benthamiana* leaves. Bar = 50 μm.

Both TargertP 2.0 and ChloroP 1.1 predicted that IbpPGM contained a chloroplast transit peptide. The expression vector pMDC83-IbpPGM was, therefore, transiently expressed in the *N. benthamiana* epidermal cells and visualized using a laser scanning confocal microscope (Figure 5). In the tobacco epidermal cells, IbpPGM was observed in scattered patches and co-localized with the autofluorescence of the chloroplasts. This result concurred with the online predictions that IbpPGM localizes to the chloroplast.

### 3.5. Overexpression of IbpPGM in the Sweet Potato

**Figure 6:**
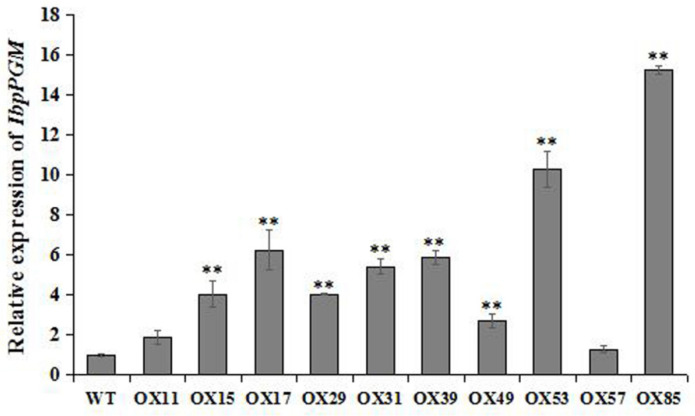
Relative expression levels of *IbpPGM* in the *IbpPGM*-overexpressing (OX) transgenic and wild-type (WT) sweet potato lines. ** indicates a significant difference versus WT at *p* < 0.01, based on Student’s *t*-test.

To functionally characterize *IbpPGM*, the recombinant vector pC3301-121-IbpPGM was introduced into the sweet potato cultivar Lizixiang. A total of 97 putative transgenic lines were obtained, and 10 lines were shown to be transgenic using GUS assays and PCR verification. The qRT-PCR indicated that the *IbpPGM* expression levels in these 10 transgenic lines were 1.3–15.3 times of the level in WT, with lines OX17, OX53, and OX85 having the three highest expression levels (Figure 6). Then, the three OX lines and the wild-type plants were further propagated in vitro and we observed that the leaf area of the in-vitro plantlets of OX was much larger than that of WT and the growth rate of OX was also faster. However, when they were grown in the field, no differences were identified between WT and OX in leaf size and shape, vine growth vigor, or morphology of the storage roots (Supplementary Figures S1 and S2). The overexpression of *IbpPGM* may have an influence on improving the photosynthesis of OX and under the lab tissue culture condition, this advantage of OX was magnified by the limited culture conditions, in terms of light, temperature, humidity, and CO_2_ concentration. When transplanted in the field, the photosynthetic ability and growth vigor of all plant lines were fully stimulated by the sufficient environmental factors and the phenotypic differences disappeared.

### 3.6. Starch and Sugar Contents in the Transgenic Sweet Potato

The quantified levels of starch and sugar contents in the transgenic and WT lines are shown in Table 2. The overexpression of *IbpPGM* in the sweet potato significantly increased the starch contents in the storage roots. Moreover, the sucrose contents in the transgenic storage roots decreased significantly, whereas the glucose and fructose contents increased.

### 3.7. Expression Profiles of the Starch Biosynthetic Genes

**Figure 7:**
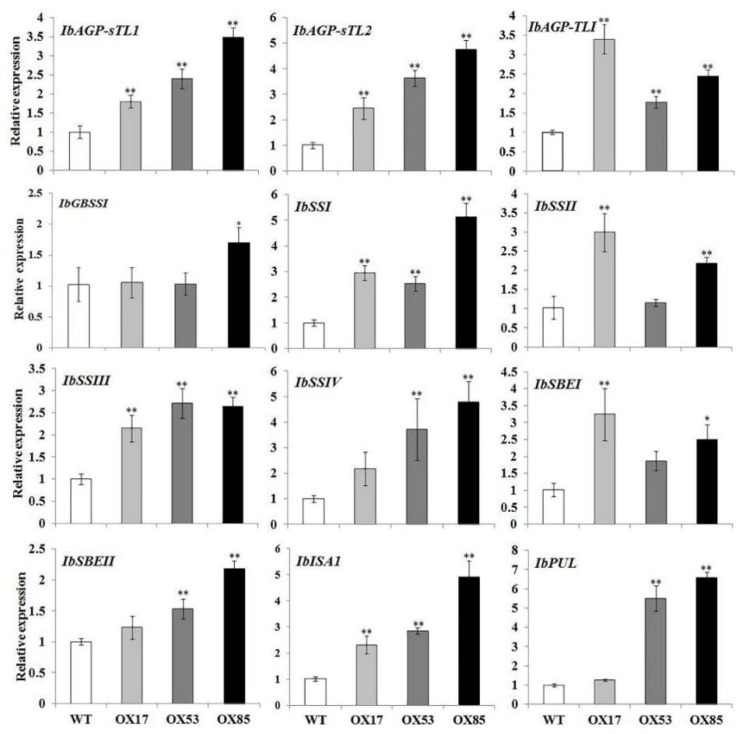
Relative expression levels of the starch biosynthesis related genes in the *IbpPGM*-overexpressing (OX) transgenic and wild-type (WT) sweet potato lines. *IbAGP-sTL1* and *-sTL2*, encoding the two small subunits of ADPglucose pyrophosphorylase (GenBank accession number: Z79635 and Z79636); *IbAGP-TLI*, encoding the large subunit of ADPglucose pyrophosphorylase (AJ252316); *IbGBSSI*, granule-bound starch synthase I (AB071604); *IbSSI*, *II*, *III* and *IV*: soluble starch synthase I, II (AF068834), III and IV; *IbSBEI* and *II*, starch branching enzyme I and II (AB194725 and AB071286); *IbISA1*, isoamylase1 (DQ074643); *IbPUL*, pullulanase. * and ** indicate a significant difference versus WT at *p* < 0.05 and <0.01, respectively, based on Student’s *t*-test.

The expression levels of the starch biosynthesis related genes in the transgenic lines were evaluated by the qRT-PCR (Figure 7). All 12 detected genes were located downstream of *IbpPGM* in the starch biosynthesis pathway and showed increased expression levels, to different extents, in the transgenic lines, compared with WT. Among the 12 genes, *IbAGP-sTL1*, *IbAGP-sTL2,* and *IbAGP-TLI* were responsible for the ADPG synthesis. *IbGBSSI* was involved in the amylose elongation, and the other eight genes were mainly involved in the amylopectin synthesis. Thus, the overexpression of *IbpPGM* promoted the accumulation of the starch biosynthesis precursors and resulted in the up-regulation of related downstream genes.

## 4. Discussion

The sweet potato is an important food crop with starchy storage roots that are raw materials for food, feed, and industrial uses. Traditional hybrid breeding results in a slow crop improvement rate and is not trait specific. Consequently, genetic engineering has become an effective way to increase the starch contents in the sweet potato storage roots, which is a primary goal of sweet potato breeding. The main functional genes in the sweet potato starch synthesis have been characterized. The overexpression of *IbAATP*, which is responsible for transporting ATP in the cytoplasm to plastids as an energy supply for starch synthesis, and *IbSSI*, which is involved in elongating the short chains of amylopectin, significantly increases the starch contents in the sweet potato storage roots [32,33]. Additionally, the overexpression of *IbSnRK1* in the sweet potato improves the starch content, as well as the starch quality, in the storage roots [36].

In the plant starch synthetic pathway, PGM is responsible for the synthesis of the upstream precursor G1P. Functional studies of PGM have mainly focused on how the starch contents in the plants changed when this gene is mutated or subject to RNA interference [10,12,15,24]. However, few studies have investigated how the starch contents in plants, including the sweet potato, are altered when PGM is overexpressed. In this study, the coding and genomic sequences of *IbpPGM* were isolated from the Xu 781 sweet potato line using homologous cloning. A GSDS analysis showed that *IbpPGM* shared an identical gene structure (22 exons and 21 introns) with its counterparts from other species, such as *Brassica napus* and *A. thaliana*, whereas *cPGM*s shared a similar gene structure of 18 exons and 17 introns. This demonstrated that the functional differentiation of the *PGM* genes into plastidial and cytosolic forms was accompanied by gene structural changes. Additionally, the deletion of the chloroplast transit peptides from the cPGM proteins, compared with the pPGM proteins, also indicated that the gene function is closely related to the gene structure.

The expression level of *IbpPGM* was high in storage roots and leaves, which was consistent with its characterized role in starch synthesis. *IbpPGM* exists in amyloplasts in storage roots, whereas in leaves, it is mainly found in chloroplasts. Sucrose is an important signaling molecule in starch synthesis, and it induces the expression of starch synthesis-related genes through the abscisic acid pathway. When the sucrose contents in leaves (or in-vitro sucrose feeding) exceeds the need for respiration, starch synthesis is induced [37,38]. In sweet potato, the expression levels of *IbGBSSI*, *IbAGP-sTL1,* and *IbSSI* are all significantly induced by exogenous sucrose treatments [26,33,39]. In this study, the leaf-petiole cuttings were soaked in water in darkness as the starvation pretreatment to consume the endogenous carbohydrates and minimize respiration. The 0 h−6 h sucrose treatment was the recovery period for respiration, during which the *IbpPGM* expression was induced at a low level. When the continuous sucrose feeding exceeded the respiratory demand, the *IbpPGM* expression began to increase rapidly, reaching 28 times the 0 h level by the end of the treatment. However, the continuous feeding of water after the starvation pre-treatment did not induce the *IbpPGM* expression. In agronomic practices, spraying sucrose-based polymers on leaves may improve the yield and quality of some fruits, which may result from induced alterations in the carbohydrate metabolism in leaves and then fruits. Thus, determining whether a leaf spray of sugar-based growth regulators to increase the yield and quality of the sweet potato, would be of interest in the future.

To determine whether *IbpPGM* encodes a mature protein, two recombinant vectors for the prokaryotic expression were constructed. One carried the complete coding sequence of *IbpPGM*, and the other carried the same sequence minus the chloroplast transit peptide (Δ*IbpPGM*). The former could not be expressed in *E. coli*, whereas the latter expressed a mature protein. Thus, the transit peptide of IbpPGM may form a complex secondary structure at the mRNA level that affects the initial translation in a prokaryotic system. The fluorescence of the GFP protein fused with the complete coding sequence of *IbpPGM* mainly targeted the chloroplasts, which indicated that the transit peptide was successfully translated in the eukaryotic system and directed the IbpPGM protein to the chloroplasts.

To characterize the function of *IbpPGM*, it was overexpressed in the sweet potato low-starch cultivar Lizixiang, by infecting the embryogenic suspension cells, and 10 positive transgenic lines were obtained. The three lines having the highest *IbpPGM* expression levels exhibited significant 4.5%, 5.5%, and 12.0% increases in the storage-root starch content, compared with the WT line. Owing to the overexpression of *IbpPGM*, the precursor G1P accumulated, which up-regulated the downstream genes involved in amylose and amylopectin synthesis, leading to further increases in the starch contents (Figure 7). In heterotrophic organs, the catalytic substrate of pPGM for G1P is G6P, which is mainly derived from the degradation of sucrose and is transported into the amyloplasts from the cytoplasm [40]. In this study, the overexpression of *IbpPGM* consumed more G6P for the G1P synthesis, which accelerated the degradation of sucrose in the cytoplasm, leading to further increases in the glucose and fructose contents.

## 5. Conclusions

In the present study, the *IbpPGM* gene was cloned from the Xu 781 sweet potato line. *IbpPGM* was mainly expressed in the storage roots and leaves. Its expression was strongly induced by the exogenous sucrose treatments. The IbpPGM protein was subcellularly localized to the chloroplasts and was successfully expressed in *E. coli* when its 73-aa chloroplastic transit peptide was excised. The overexpression of *IbpPGM* significantly increased the starch contents of the transgenic sweet potato storage root and altered its soluble sugar levels. Meanwhile, the expression levels of starch biosynthetic genes in transgenic sweet potato storage roots showed increased expression levels to different extents. These results indicated that *IbpPGM* has the great potential as an important candidate gene for increasing the starch content of the sweet potato through genetic engineering.

## Figures and Tables

**Table 1:** Primers used in this study.

Name	Sequence (5′-3′)	Name	Sequence (5′-3′)
DF	AGCCAYAAYCCWGGTGGWCC	DR	CCRTAWGTVGCCCARTRCT
GF	ACACAACACAACACACACTCTTCT	GR	GCGCAGACAAAATTATACAGATT
qPGM-F	TCAAGCACTCAAGATTAAATCGGTT	qPGM-R	ATCACCTCCGAGAACCAACAAC
Actin-F	AGCAGCATGAAGATTAAGGTTGTAGCAC	Actin-R	TGGAAAATTAGAAGCACTTCCTGTGAAC
pET-F (*Nco* I)	CATGCCATGGGGGCGTCGTTTTGTGCG	pET-R (*Xho* I)	CCCTCGAGTGTTATGACAGTTGGCTTCTC
pET-ΔF (*Nco* I)	CATGCCATGGGGGCTACCGTCGCCGAATC		
83-F (*Pac* I)	CCTTAATTAAATGGCGTCGTTTTGTGC	83-R (*Asc* I)	AGGCGCGCCATGTTATGACAGTTGGCTTCTCTCT
qIbAGP-sTL1-F	AGAGAATTGACGGTGATGTTAGCA	qIbAGP-sTL1-R	ATGAACGGAGCAGTCCGAAC
qIbAGP-sTL2-F	CCAAAAGGAGAACAGTTGAAAGCTA	qIbAGP-sTL2-R	CTCCAGGGAACTTTTCTCGAAGTA
qIbAGP-TLI-F	GAGATATCCCACATCCAACGACTT	qIbAGP-TLI-R	TAGGGCCAAGTTAGCGTCGTAG
qIbGBSSI-F	TGGCAACTATAACTGCCTCACAC	qIbGBSSI-R	GGCACTGGTTCTCAATTGTAACAT
qIbSSI-F	GCTGCAGACCGTCTTTGTGC	qIbSSI-R	GAGCCATCCCTCTGTGCTCC
qIbSSII-F	AGACTGTGGGATCTACTGAAAGGC	qIbSSII-R	GTGAATCCACGTCCAGTGGC
qIbSSIII-F	TCTGTTATCCTGAGGAGGTAAAACC	qIbSSIII-R	CTCCCATGATCAATACATCAGGC
qIbSSIV-F	CTGCTTTCTCATTTCTGTCATCGT	qIbSSIV-R	GCTCAACTTCCACTTGACTCAGAG
qIbSBEI-F	ATTCTTGGCCTAGACCAAGGG	qIbSBEI-R	ACAATGCAGCCTTCTTCTTTGTTA
qIbSBEII-F	AGTCCGCTGTTTGGAGGCTT	qIbSBEII-R	CCTCAACTGGTTTTGCTTCGTC
qIbISA1-F	GGAACGAGGTGGTTATCGGTG	qIbISA1-R	TCTGGGCATAGCAACAGAATTATG
qIbPUL-F	GCTGCTCGACGATGCCTCT	qIbPUL-R	CATCCTCAACGTCCACATTCC

Primers starting with “q” are used for the qRT-PCR. The underlined nucleotides are restriction enzyme sites.

**Table 2:** Starch and soluble sugar contents in the storage roots of the plastidial phosphoglucomutase-overexpressing (OX) transgenic and wild-type (WT) sweet potato lines.

Lines	Starch (% of DW)	Sucrose (mg 100 g^−1^ DW)	Glucose (mg 100 g^−1^ DW)	Fructose (mg 100 g^−1^ DW)
WT	58.07 + 0.32	15.14 + 0.11	0.39 + 0.05	0.18 + 0.03
OX17	60.69 + 1.69 *	14.19 + 0.09 **	1.22 + 0.02 **	0.63 + 0.01 **
OX53	61.25 + 1.75 *	13.71 + 0.13 **	0.81 + 0.03 **	1.39 + 0.02 **
OX85	65.05 + 0.97 **	11.95 + 0.04 **	0.57 + 0.01 **	0.24 + 0.02 **

* and ** indicate a significant difference versus WT at *p* < 0.05 and <0.01, respectively, based on Student’s t-test.

## Data Availability

Not applicable.

## Supplementary Materials

The following supporting information can be downloaded at: https://www.mdpi.com/article/10.3390/genes13122234/s1, Figure S1: Production of the transgenic sweet potato plants overexpressing the *IbpPGM* gene. (a) Proliferation of embryogenic suspension cultures of cultivar Lizixiang in MS medium containing 2.0 mg L-1 2,4-D. (b) Phosphinothricin (PPT)-resistant calluses (bright yellow) formed after 8 weeks of selection on MS medium containing 2.0 mg L-1 2,4-D, 300 mg L-1 cefotaxime sodium and 50 mg L-1 PPT. (c) Regeneration of plantlets from PPT-resistant calluses on MS medium containing 1.0 mg L-1 ABA and 300 mg L-1 cefotaxime sodium. (d) Positive and negative GUS staining of the leaves of OX and WT plants, respectively. (e) PCR analysis of GUS positive OX lines. Lane M: BL2000 DNA marker; Lane W: water as a negative control; Lane P: plasmid pC3301-121-IbpPGM as a positive control; Lane WT: WT as a negative control; Lanes 11-85: GUS positive OX lines. (f) WT and OX plants grown in the field. (g) Storage roots of the WT and OX plants. Figure S2: Differences in leaf area and growth rate between the in-vitro plantlets of WT and OX lines.
