# Supplementary material for: Plastidial Phosphoglucomutase (pPGM) Overexpression Increases the Starch Content of Transgenic Sweet Potato Storage Roots
Source: Genes (Basel). 2022 Nov 28;13(12):2234. doi: 10.3390/genes13122234 (PMC9778278; doi:10.3390/genes13122234)
Supplement: Supplementary file 1 [file genes-13-02234-s001.zip › Supplementary Figures.pdf]

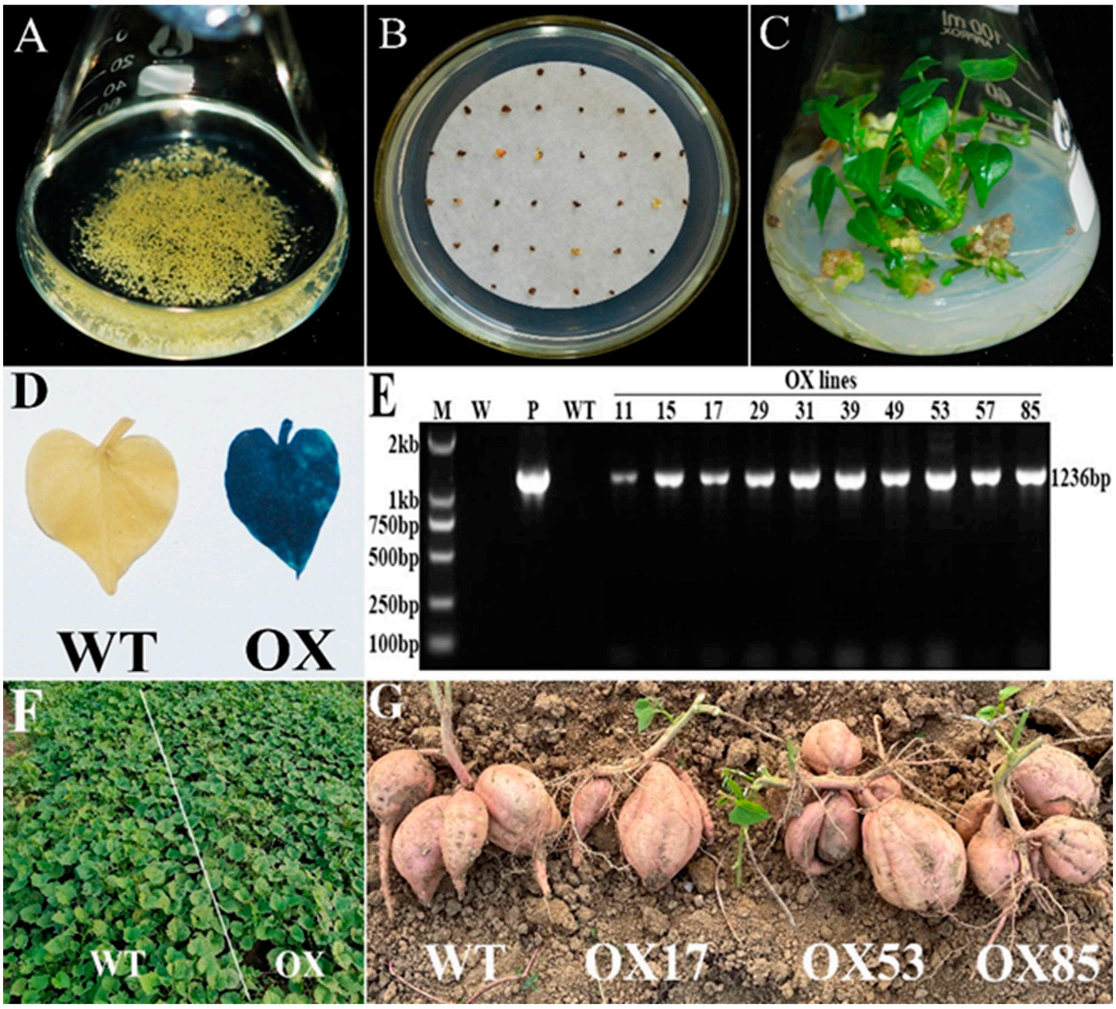

Supplementary Figure S1. Production of the transgenic sweet potato plants overexpressing the *IbpPGM* gene. (a) Proliferation of embryogenic suspension cultures of cultivar Lizixiang in MS medium containing 2.0 mg L^{-1} 2,4-D. (b) Phosphinothricin (PPT)-resistant calluses (bright yellow) formed after 8 weeks of selection on MS medium containing 2.0 mg L^{-1} 2,4-D, 300 mg L^{-1} cefotaxime sodium and 50 mg L^{-1} PPT. (c) Regeneration of plantlets from PPT-resistant calluses on MS medium containing 1.0 mg L^{-1} ABA and 300 mg L^{-1} cefotaxime sodium. (d) Positive and negative GUS staining of the leaves of OX and WT plants, respectively. (e) PCR analysis of GUS positive OX lines. Lane M: BL2000 DNA marker; Lane W: water as a negative control; Lane P: plasmid pC3301-121-IbpPGM as a positive control; Lane WT: WT as a negative control; Lanes 11-85: GUS positive OX lines. (f) WT and OX plants grown in the field. (g) Storage roots of the WT and OX plants.

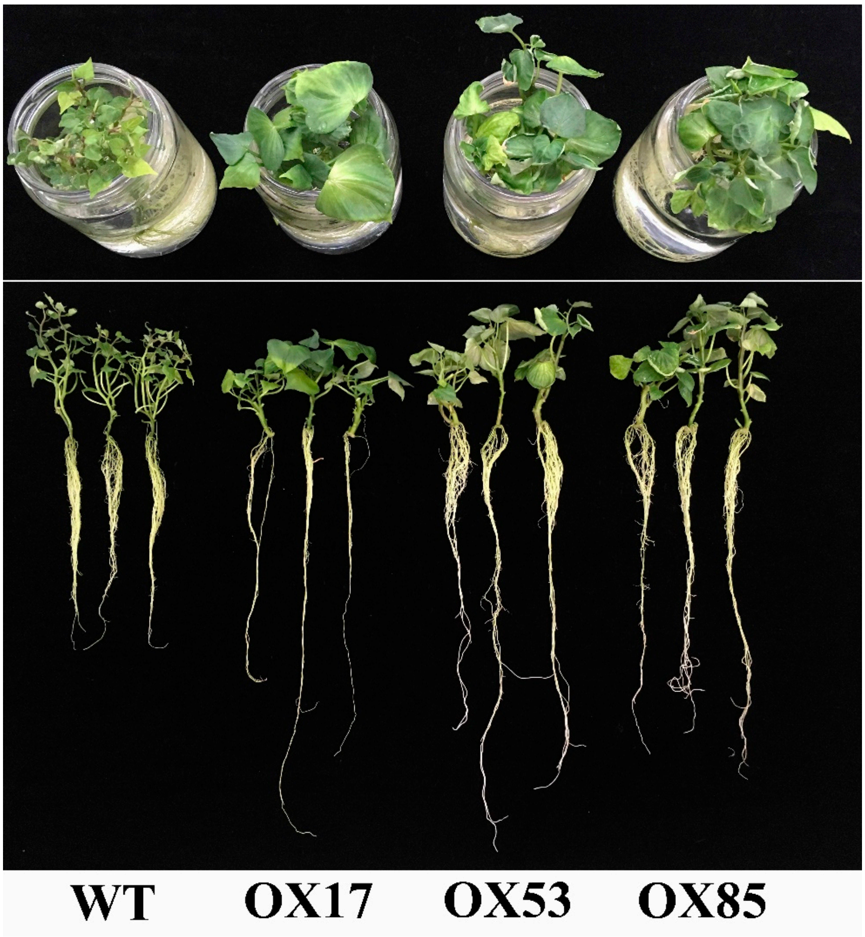

Supplementary Figure S2. Differences in leaf area and growth rate between the in-vitro plantlets of WT and OX lines.
