# Supplementary figures and images for: Plastidial Phosphoglucomutase (pPGM) Overexpression Increases the Starch Content of Transgenic Sweet Potato Storage Roots
Source: Genes (Basel). 2022 Nov 28;13(12):2234. doi: 10.3390/genes13122234 (PMC9778278; doi:10.3390/genes13122234)

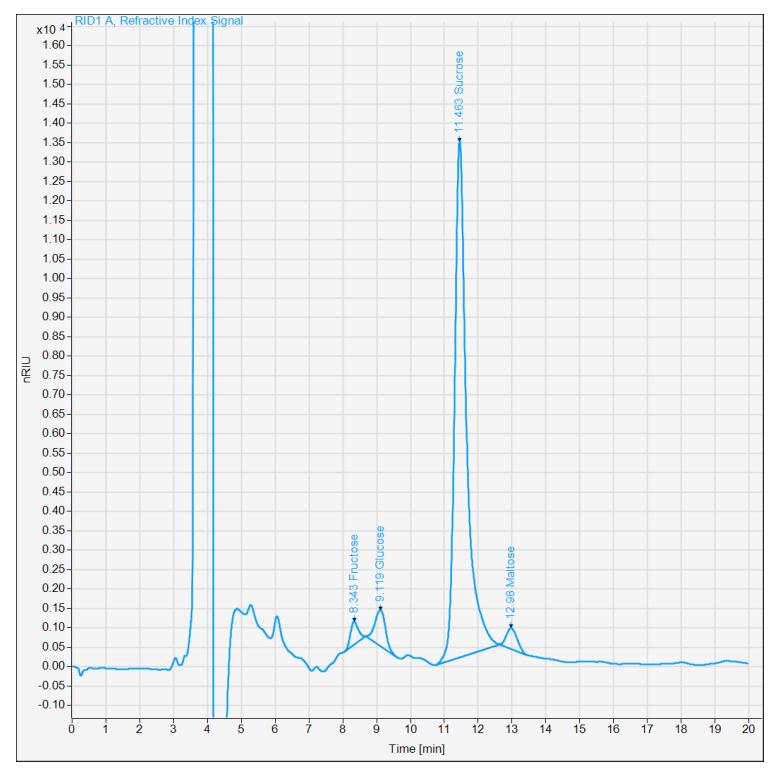

Supplement: Supplementary file 1 [file genes-13-02234-s001.zip › Chromatography of all samples/OX17-1.jpg]

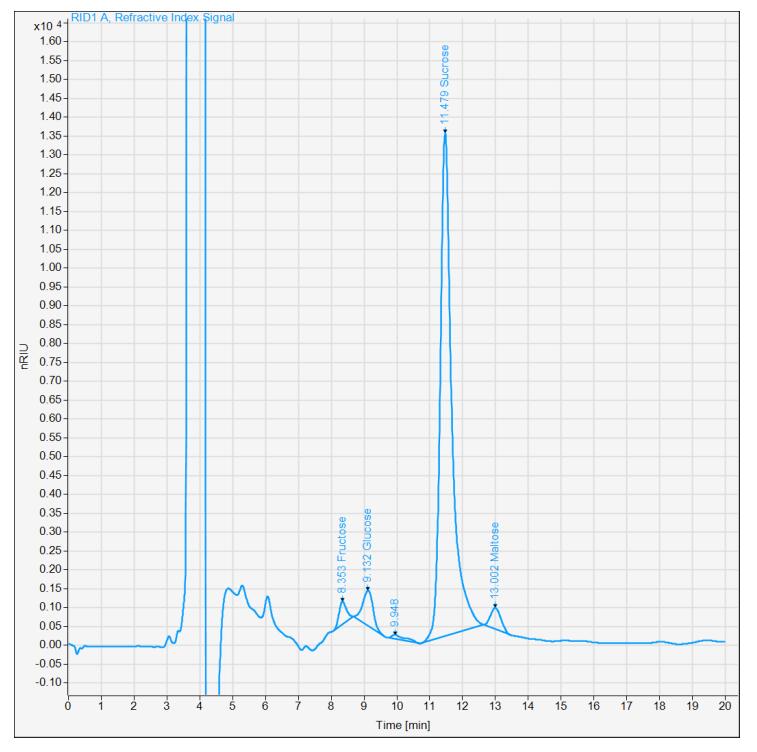

Supplement: Supplementary file 1 [file genes-13-02234-s001.zip › Chromatography of all samples/OX17-2.jpg]

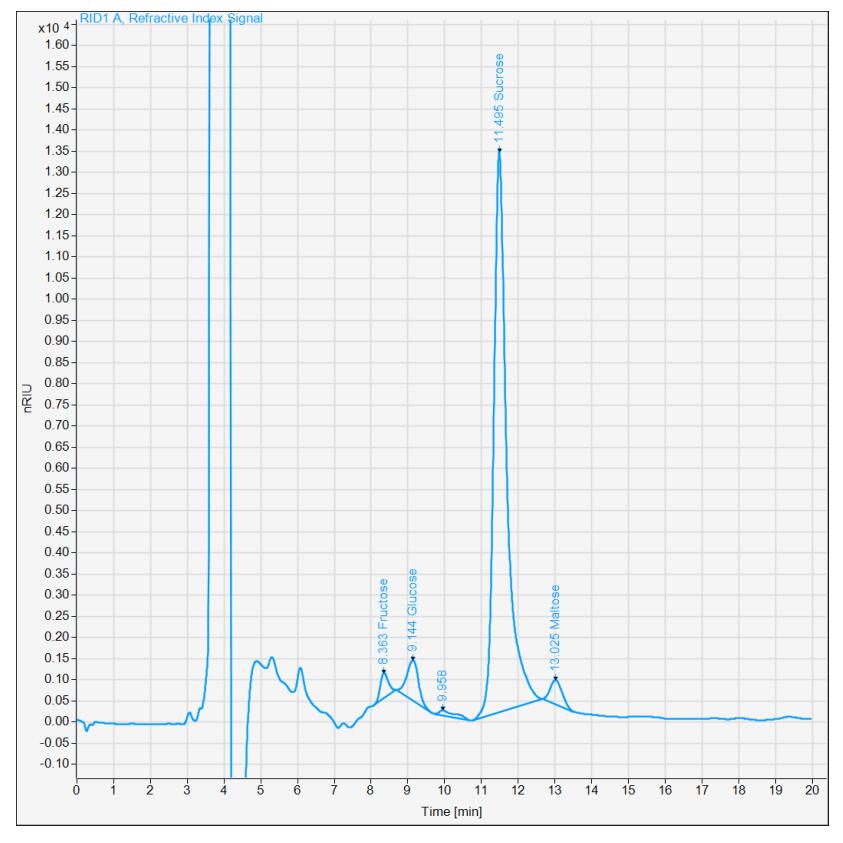

Supplement: Supplementary file 1 [file genes-13-02234-s001.zip › Chromatography of all samples/OX17-3.jpg]

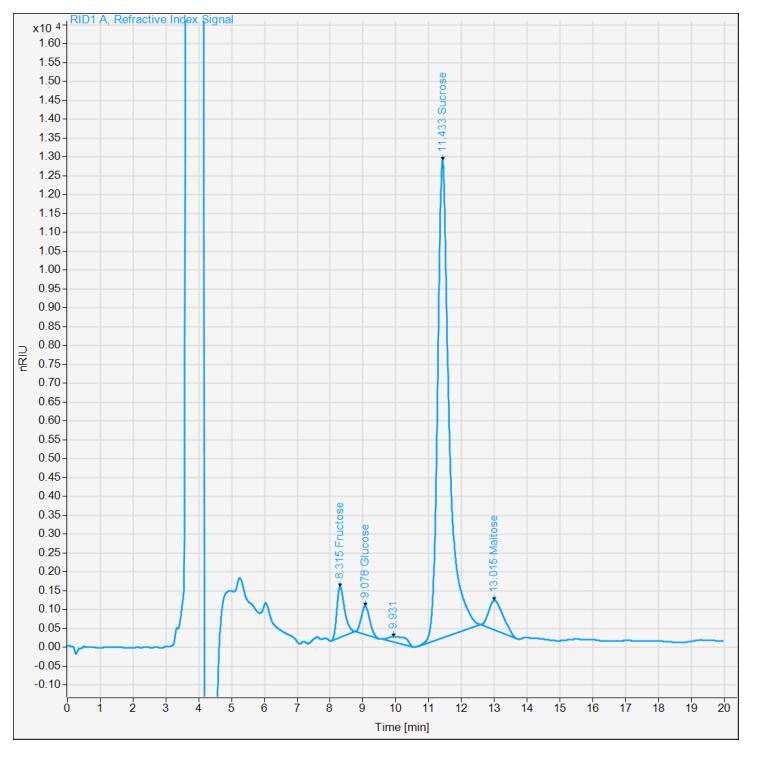

Supplement: Supplementary file 1 [file genes-13-02234-s001.zip › Chromatography of all samples/OX53-1.jpg]

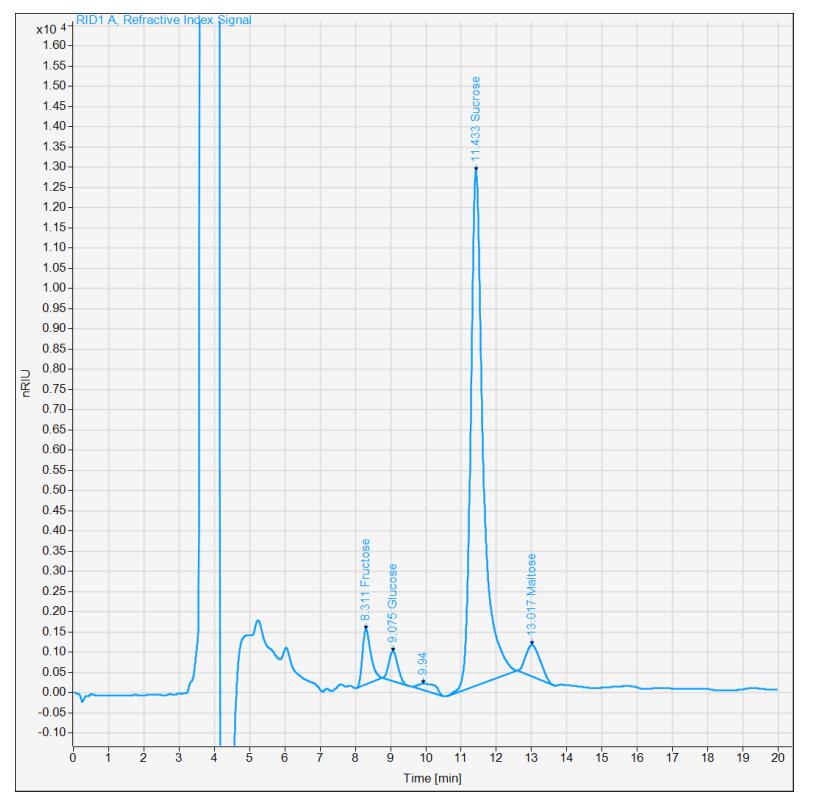

Supplement: Supplementary file 1 [file genes-13-02234-s001.zip › Chromatography of all samples/OX53-2.jpg]

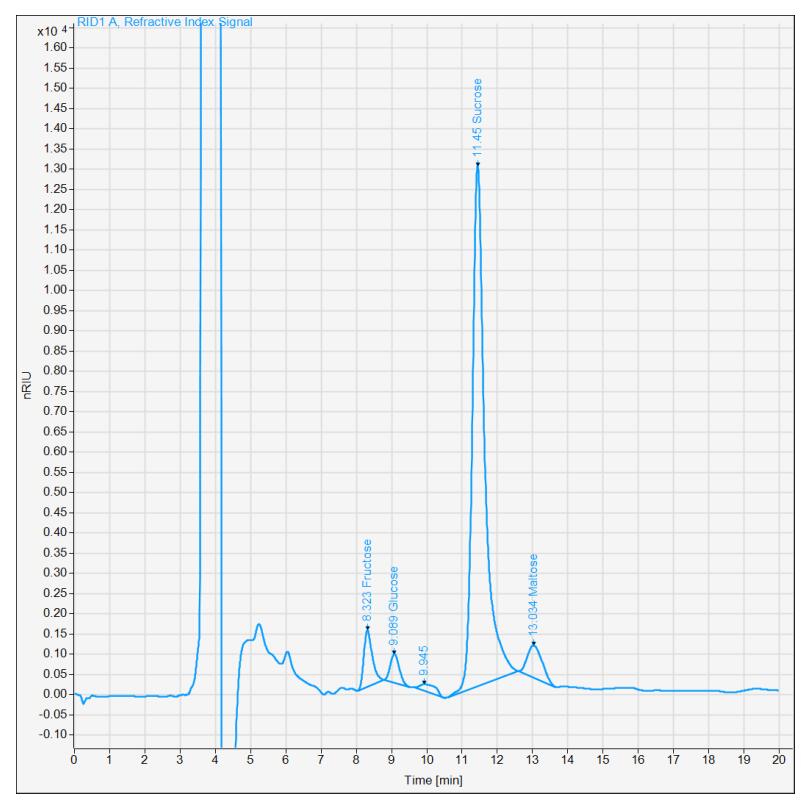

Supplement: Supplementary file 1 [file genes-13-02234-s001.zip › Chromatography of all samples/OX53-3.jpg]

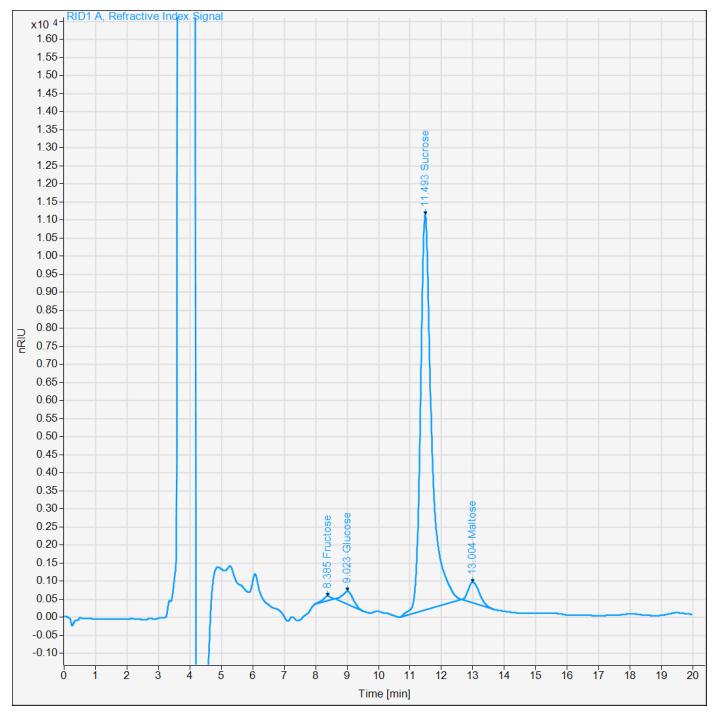

Supplement: Supplementary file 1 [file genes-13-02234-s001.zip › Chromatography of all samples/OX85-1.jpg]

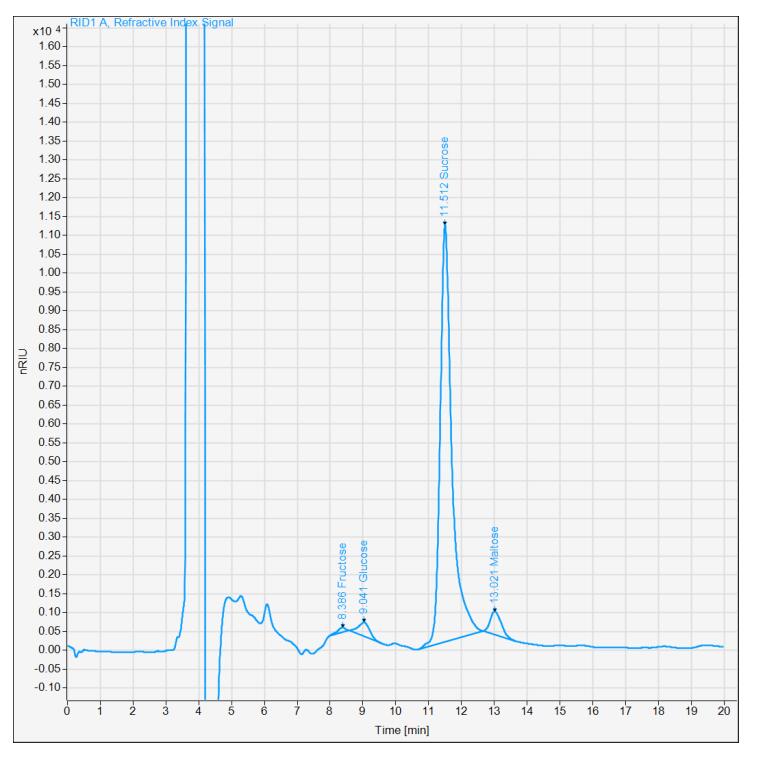

Supplement: Supplementary file 1 [file genes-13-02234-s001.zip › Chromatography of all samples/OX85-2.jpg]

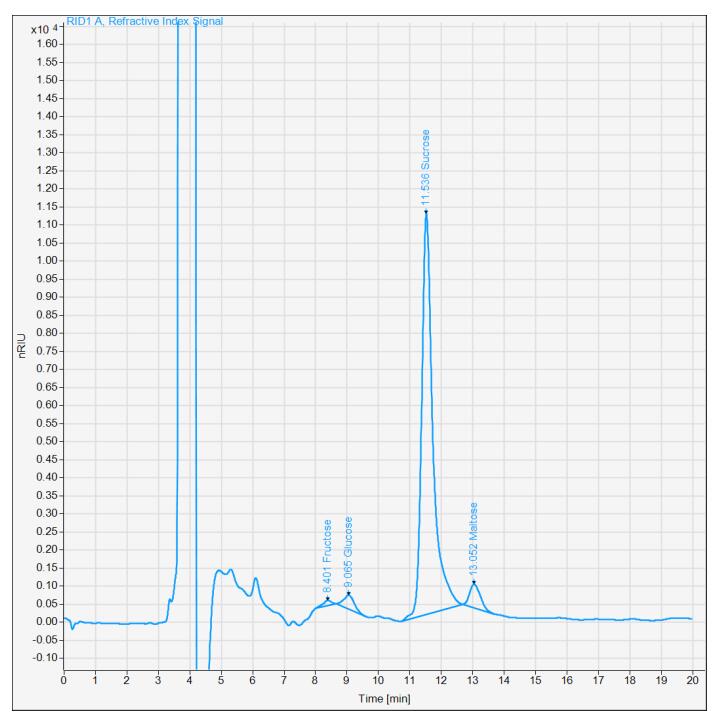

Supplement: Supplementary file 1 [file genes-13-02234-s001.zip › Chromatography of all samples/OX85-3.jpg]

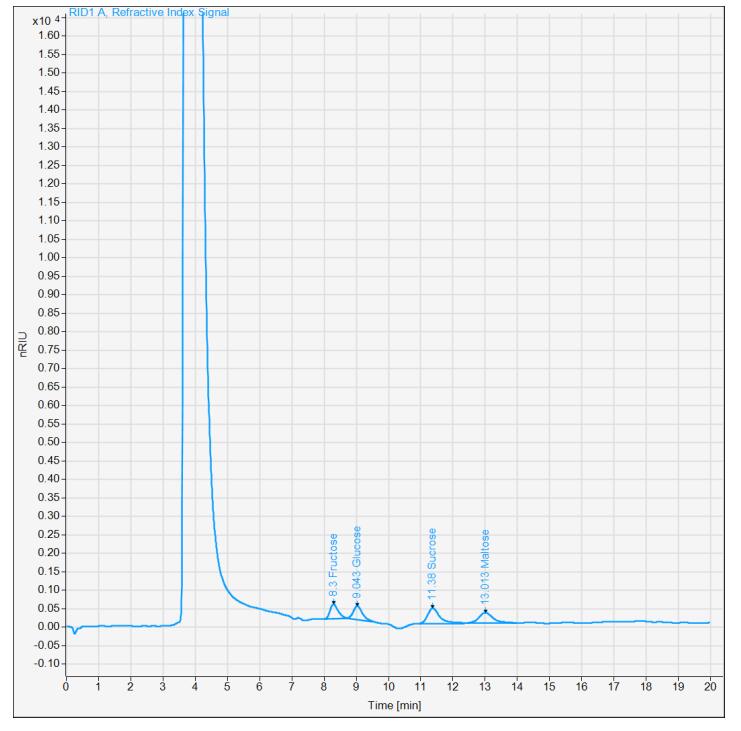

Supplement: Supplementary file 1 [file genes-13-02234-s001.zip › Chromatography of all samples/standard mix 0.1.jpg]

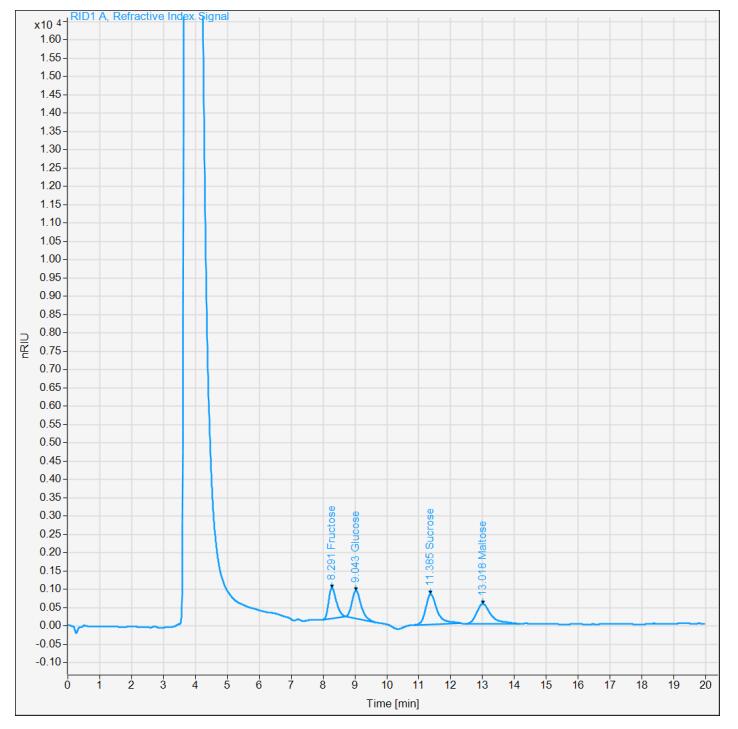

Supplement: Supplementary file 1 [file genes-13-02234-s001.zip › Chromatography of all samples/standard mix 0.2.jpg]

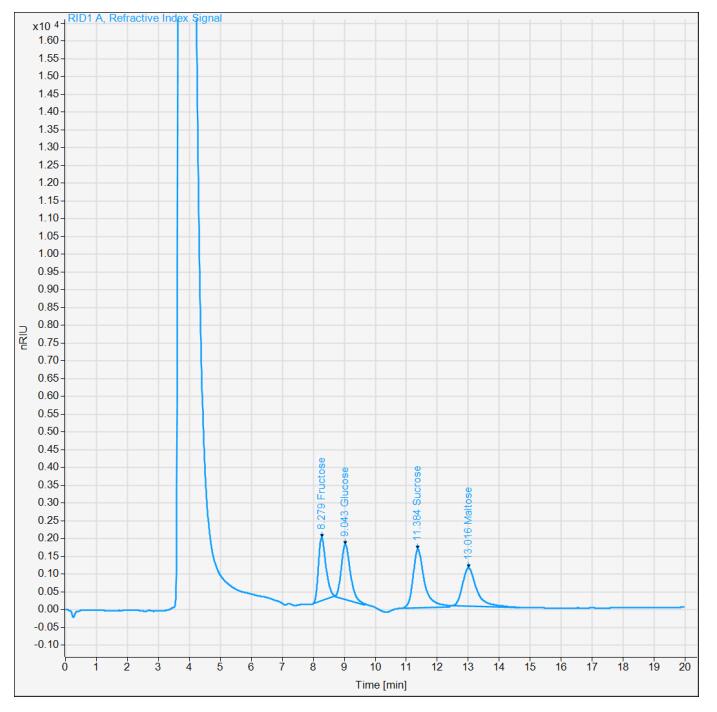

Supplement: Supplementary file 1 [file genes-13-02234-s001.zip › Chromatography of all samples/standard mix 0.4.jpg]

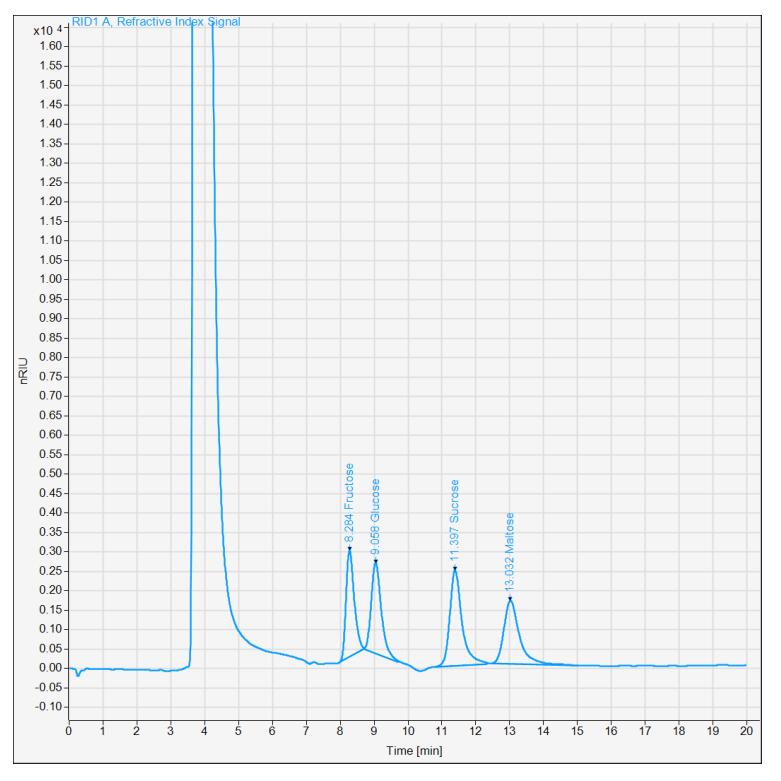

Supplement: Supplementary file 1 [file genes-13-02234-s001.zip › Chromatography of all samples/standard mix 0.6.jpg]

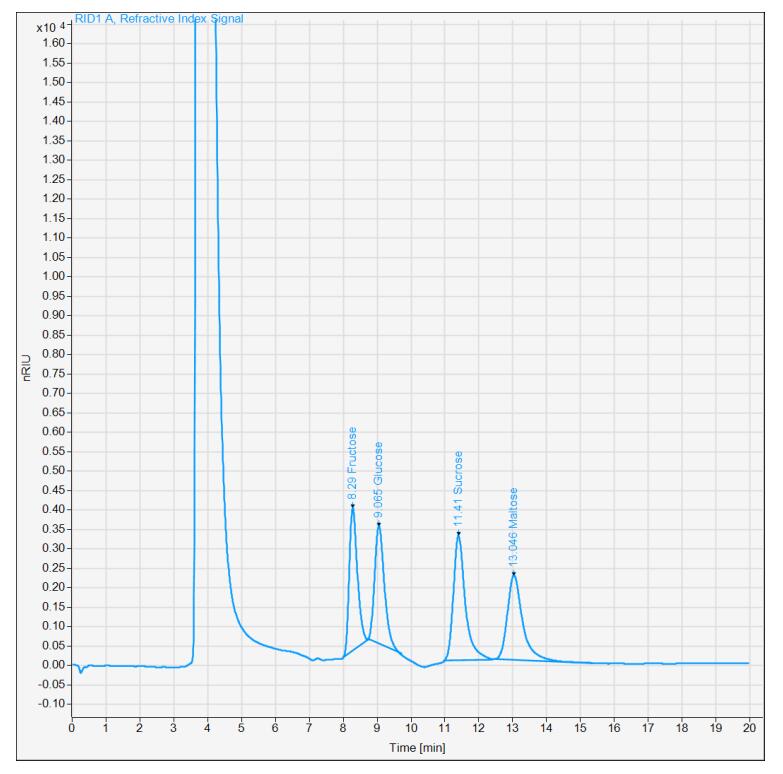

Supplement: Supplementary file 1 [file genes-13-02234-s001.zip › Chromatography of all samples/standard mix 0.8.jpg]

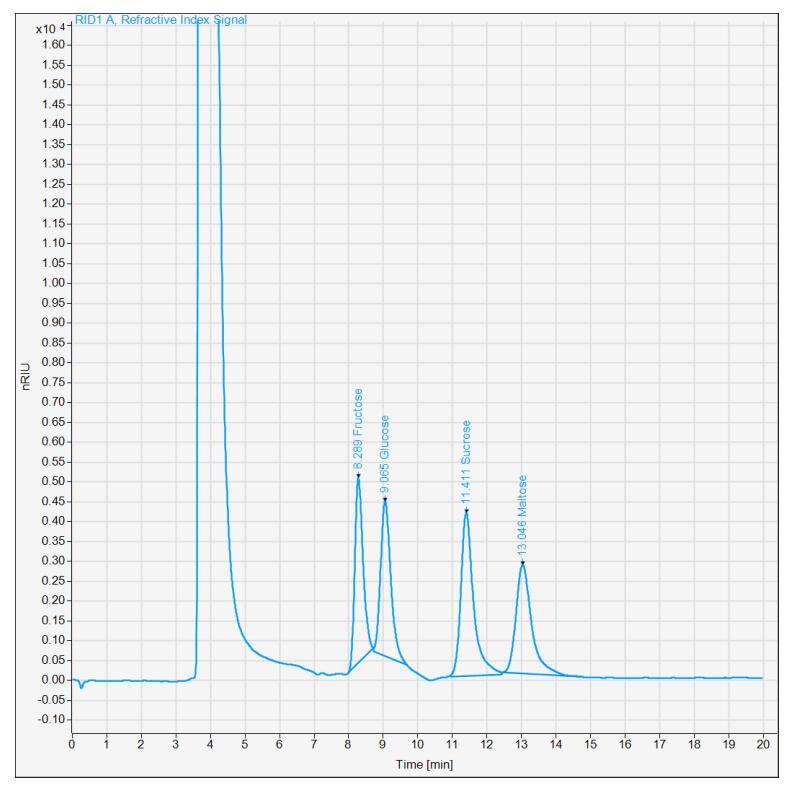

Supplement: Supplementary file 1 [file genes-13-02234-s001.zip › Chromatography of all samples/standard mix 1.0.jpg]

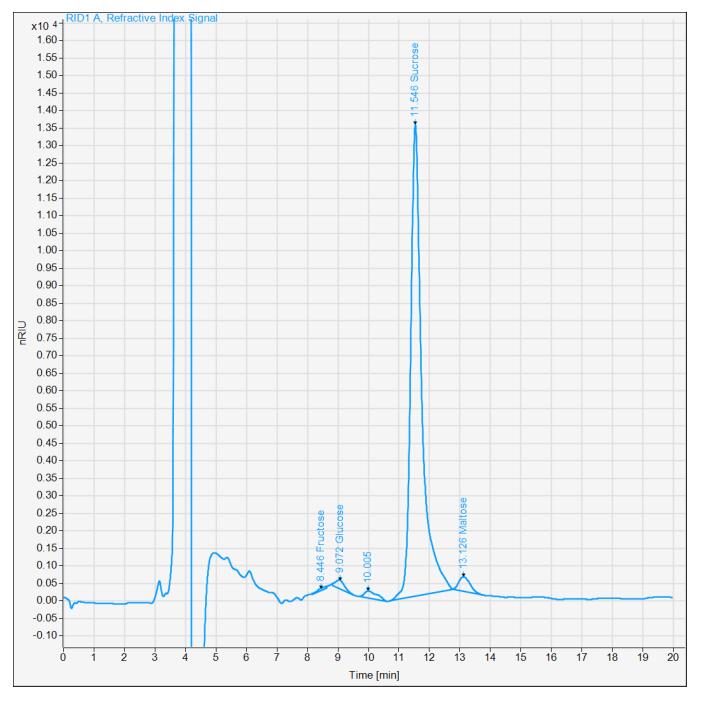

Supplement: Supplementary file 1 [file genes-13-02234-s001.zip › Chromatography of all samples/WT-1.jpg]

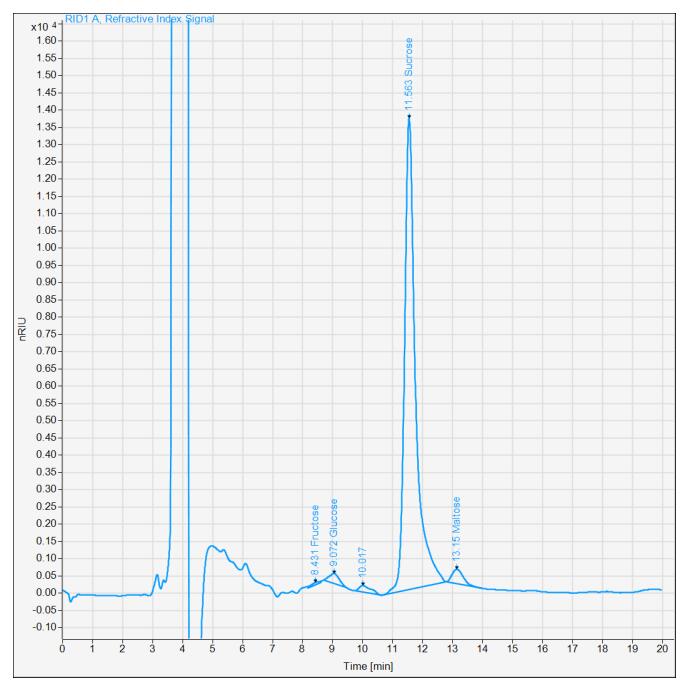

Supplement: Supplementary file 1 [file genes-13-02234-s001.zip › Chromatography of all samples/WT-2.jpg]

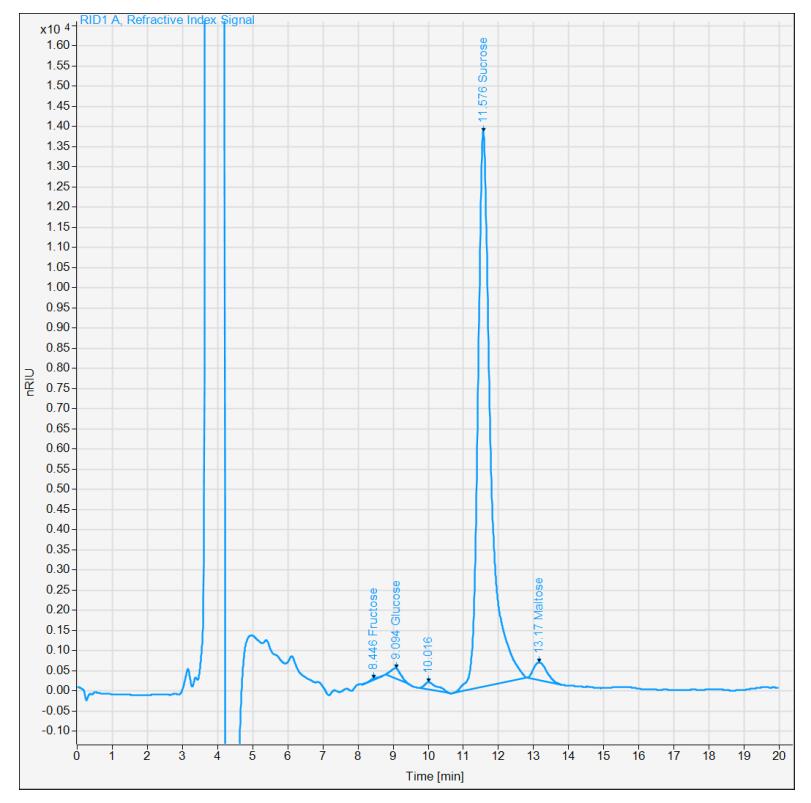

Supplement: Supplementary file 1 [file genes-13-02234-s001.zip › Chromatography of all samples/WT-3.jpg]
